# The Role of Hydrogen Bonding in Paracetamol–Solvent and Paracetamol–Hydrogel Matrix Interactions

**DOI:** 10.3390/ma14081842

**Published:** 2021-04-08

**Authors:** Marta Miotke-Wasilczyk, Marek Józefowicz, Justyna Strankowska, Jerzy Kwela

**Affiliations:** Insitute of Experimental Physics, Faculty of Mathematics, Physics and Informatics, University of Gdańsk, Wita Stwosza 57, 80-308 Gdańsk, Poland; justyna.strankowska@ug.edu.pl (J.S.); jerzy.kwela@ug.edu.pl (J.K.)

**Keywords:** paracetamol, drug release, hydrogel matrix, hydrogen bond, solvatochromism, solvent effects

## Abstract

The photophysical and photochemical properties of antipyretic drug – paracetamol (PAR) and its two analogs with different substituents (acetanilide (ACT) and *N*-ethylaniline (NEA)) in 14 solvents of different polarity were investigated by the use of steady–state spectroscopic technique and quantum–chemical calculations. As expected, the results show that the spectroscopic behavior of PAR, ACT, and NEA is highly dependent on the nature of the solute–solvent interactions (non-specific (dipole-dipole) and specific (hydrogen bonding)). To characterize these interactions, the multiparameter regression analysis proposed by Catalán was used. In order to obtain a deeper insight into the electronic and optical properties of the studied molecules, the difference of the dipole moments of a molecule in the ground and excited state were determined using the theory proposed by Lippert, Mataga, McRae, Bakhshiev, Bilot, and Kawski. Additionally, the influence of the solute polarizability on the determined dipole moments was discussed. The results of the solvatochromic studies were related to the observations of the release kinetics of PAR, ACT, and NEA from polyurethane hydrogels. The release kinetics was analyzed using the Korsmayer-Peppas and Hopfenberg models. Finally, the influence of the functional groups of the investigated compounds on the release time from the hydrogel matrix was analyzed.

## 1. Introduction

One of the main goals of pharmaceutical research is to improve the pharmacokinetic and pharmacodynamic properties of medicinal products [1]. Despite the great potential of modern pharmaceuticals, their low solubility results in poor oral bioavailability [2,3]. To overcome these limitations, polymeric drug carriers are used in which the poorly soluble active substance is dispersed [4,5,6,7]. As was shown, the polymer can decrease the drug crystallization ability due to polymer-drug interactions (such as hydrogen bonding) by reducing drug mobility and limiting drug-drug molecular interactions [8,9,10]. Thus, intermolecular interactions between the drug and the polymer can improve the drug stability, and consequently its bioavailability. Hence, the presence of hydrogen bonding between the carrier and the drug is the key to construct an efficient system in terms of controlled drug release [11,12,13].

Despite the growing interest in understanding drug-carrier interactions at a molecular level, prediction of the drug release kinetics remains challenging due to many interrelated physicochemical processes that occur simultaneously in the aqueous environment [14,15]. Therefore, studies on the interaction of active substances (drugs) and their carriers in hydrous medium are important as they provide the basic foundation for their application [16,17,18,19,20]. The influence of hydrogen bond interactions on the drug release kinetics need to be determined in order to understand the fundamental mechanisms governing the release process [21,22].

The main goal of the current work was to investigate the role of hydrogen bonding in all drug-polymer and drug-solvent interactions in order to identify the effect of these interactions on the active substance (paracetamol (PAR), acetanilide (ACT), and *N*-ethylaniline (NEA)) release process from polymer carrier (polyurethane hydrogel matrix). Polyurethane hydrogel matrices are commonly used as the drug carriers due to their biocompatibility and the ability to retain biologically active agents in a water-swollen networks [23]. Paracetamol (acetaminophen, PAR) due to its anti-inflammatory, analgesic and antipyretic properties is one of the most popular and widespread active substance [24,25]. To determine the role of the chemical structure of the active substance (different substituents) on the kinetics of the release process, two substances of similar structure, but differing in the presence of functional groups with hydrogen-bonding ability, were also studied (acetanilide (ACT) and *N*-ethylaniline (NEA)).

This paper presents research on the determination of two types of interactions (non-specific and specific) between active substance and different environments, i.e., a liquid medium and a hydrogel matrix. For this purpose, we proposed a combination of solvatochromic studies in liquid system and drug release studies from the hydrogel matrix. At first, we used the steady-state absorption and emission spectroscopy to investigate solvent-dependent photophysical characteristics. The linear solvation energy dependence (LSER) analysis proposed by Catalán [26] was used to distinguish between non-specific (dipole-dipole) and specific (hydrogen bond) interactions in liquid systems. Considering the fact, that (**i**) one of the important physical parameters of molecules, which describe the distribution of electrons around them, are dipole moments in the ground and excited states, (**ii**) the knowledge of the charge distribution and the dipole moments values is important to understanding the physico-chemical processes in bulk solution, (**iii**) the solute polarizability can affect the ground and excited state dipole moments, (**iv**) the solute-solvent interactions can be also described in terms of the changes in dipole moment of fuorophore upon excitation, we used the theoretical models proposed by Lippert, Mataga, McRae, Bakhshiev, Bilot and Kawski to determine the differences between dipole moments in the ground and excited states [27,28,29,30,31]. In addition, we examined the influence of solute polarizability on dipole moments. The results of the solvatochromic studies were related to the observations of the release kinetics of PAR, ACT, and NEA from polyurethane hydrogels and were analyzed based on the Korsmayer-Peppas and Hopfenberg theoretical models [32,33]. These findings allowed to determine the influence of the chemical structure of the active substance on the release time from the hydrogel matrix.

We showed that the hydrogen bonding studies are important for understanding the study of the release process of active substances.

## 2. Experimental Section

### 2.1. Materials

**Figure 1:**
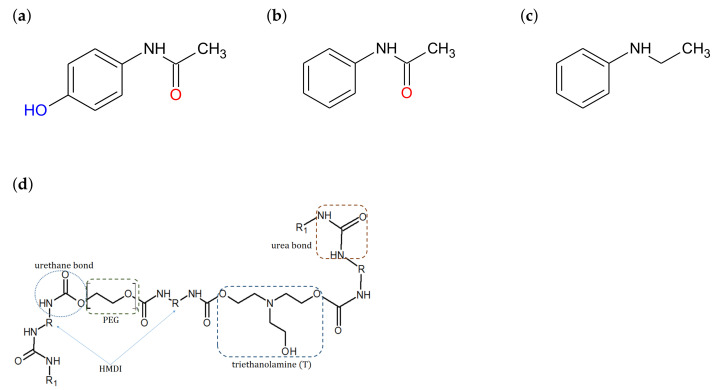
Chemical structure of (**a**) paracetamol (PAR), (**b**) acetanilide (ACT), (**c**) *N*-ethylaniline (NEA), and (**d**) fragment of the structure of the polyurethane hydrogel matrix chain.

Paracetamol (PAR, acetaminophen, *N*-(4-hydroxyphenyl)acetamide), acetanilide (ACT, *N*-phenylacetamide) and *N*-ethylaniline (NEA, monoethylaniline) (see Figure 1a–c) were purchased from Sigma-Aldrich, Inc. (Steinheim, Germany). All of the solvents used for spectroscopic studies (see Table 1) were of the highest grade, commercially available and were used without further purification.

The study of the release of PAR, ACT, and NEA into deionized water was carried out using hydrogel matrices consisting of a polyurethane and polyethylene glycol (PEG) copolymer (see Figure 1d). The hydrogel synthesis and its mechanical properties were described in our previous articles [34,35].

### 2.2. Apparatus and Methods

#### 2.2.1. Steady-State Spectroscopic Measurements and Quantum-Chemical Calculations

The absorption and emission spectra of the molecules under study were recorded using a computer-controlled spectrophotometer (UV-2401 PC, Shimadzu, Kyoto, Japan) and spectrofluorometer (RF-5301, Shimadziu, Kyoto, Japan), respectively. In order to obtain more insight into the kinetics of the release process, hydrogel sample after 24 h of swelling the solution with active substance was placed in water, and, after every 3 min, the absorption spectra were recorded. Hereby the concentration of the active substance in the water was determined. The measurements were taken until no changes in the absorption spectra were observed (until reaching the equilibrium state, when the change in the value of the molar concentration between successive measurement points did not exceed 1%). All experiments were carried out three times.

All calculations regarding spectroscopic properties of PAR, ACT, and NEA were performed using the CAChe WS 5.04 program. The geometrical structure was determined using the PM3 semiempirical molecular orbital method at the Restricted Hartree Fock (RHF) level including single excitation configuration interaction (CIS). The calculations were performed for isolated molecules (gas phase).

#### 2.2.2. Multiparametric Linear Regression Analysis

In the absence of the solute–solvent specific interactions (general solvent effects), Stokes shift (the difference between absorption (a) and fluorescence (f) band positions) can be interpreted in terms of the changes in the dipole moment which occur upon excitation and the energy of a dipole in solvents of various dielectric constants (ε) and refractive index (*n*) [36,37,38,39]. However, one notices that Stokes shift is generally larger in protic solvents, in which specific interaction (e.g., hydrogen bonding) between the solute and solvent occurs. Therefore, in order to describe the solvatochromic shifts in a comprehensive way, various theoretical and empirical models were developed to differentiate between the specific and non-specific interactions [40].

One of the most widely used solvent polarity scale is the E${}_{T}$(30) scale introduced by Reichardt et al. [41]. E${}_{T}$(30) values are simply defined as the molar electronic transition energies (E${}_{T}$) of dissolved pyridinium *N*-phenolate betaine dye, measured in kilocalories per mole (kcal/mol) at room temperature (25 °C) and normal pressure (1 bar) [42]. More often, the normalized E${}_{N}^{T}$ parameter, defined according to Equation (1), is used:

(1) $$E_{N}^{T}=\frac{E_{T}\left(\mathrm{solvent}\right)-E_{T}\left(\mathrm{TMS}\right)}{E_{T}\left(\mathrm{water}\right)-E_{T}\left(\mathrm{TMS}\right)},$$

where TMS denotes tetramethylsilane. The E${}_{N}^{T}$ scale ranges from 0.000 for TMS (the least polar solvent) to 1.000 for water (the most polar solvent). Both E${}_{T}$(30) and E${}_{N}^{T}$ values can be equally used, but, for the correlation analysis of solvent effect on chemical and physical properties, the E${}_{N}^{T}$ parameter seems to be more suitable [42].

One the other hand, Kamlet and Taft [43,44] and also Catalán [45,46] developed multiparameter relationship for the description of the solvent effects (specific (acidity–basicity) and non-specific (polarity/polarizability)) on the luminescent characteristics (absorption and emission spectra, quantum yield, fluorescence lifetime etc.). In the present study, the Catalán model was chosen, because it allows to more detailed description, i.e., solvent polarity parameter has been separated by Catalán and co-workers into two independent parameters: solvent polarizability and dipolarity. This model described solvent-dependent property (*x*) in terms of reference ($x_{0}$–the statistical value of the property in the gas phase) and a linear combination of separated and complementary solvent parameters:

(2) $$x=x_{0}+s_{\mathrm{SP}}SP+s_{\mathrm{SdP}}SdP+a_{\mathrm{SA}}SA+b_{\mathrm{SB}}SB,$$

where SP and SdP parameters describe non-specific contributions regarding solvent polarizability and solvent dipolarity, respectively. The SA and SB parameters describe specific contributions regarding solvent’s hydrogen bond donor strength and hydrogen bond acceptor strength, respectively [45,46].

In Equation (2), the relative contribution of the each solvent parameter (SP, SdP, SA, SB) to spectroscopic property (*x*) can be described by the regression coefficients: $s_{\mathrm{SP}}$, $s_{\mathrm{SdP}}$, $a_{\mathrm{SA}}$, $b_{\mathrm{SB}}$. The relative ratios of these parameters allow for identification of possible types of interactions of the molecule with the microenvironment.

#### 2.2.3. Estimation of the Dipole Moments

As it was mentioned earlier, the polar molecule of the active substance in the liquid environment interacts with the solvent molecules in the process of solvation. It involves the organization of the solvent molecules around a solute molecule, resulting in a solvation shell [47]. A luminescent molecule having a constant electric dipole moment creates an electric field (reactive field) around itself. When this molecule is placed in a polar solvent, the dipole moments of the solvent molecules align along the field force lines. The solute-solvent interactions can be described in terms of its ground ($\mu_{g}$) and excited ($\mu_{e}$) state dipole moments and reactive fields around these dipoles [27,28,29,30,31,48,49].

Based on the Onsager’s description [50] of non-specific solute-solvent interactions and the Franck–Condon rule, the following formula describing the solvent induced Stokes shifts (the difference between absorption (*a*) and fluorescence (*f*) band positions) versus the solvent polarity function (f(ε,n)) can be presented as:

(3) $$\tilde{\nu_{a}}-\tilde{\nu_{f}}=m_{1}f\left(\varepsilon ,n\right)+\mathrm{const}.,$$

where

(4) $$m_{1}=\frac{2{\left(\vec{\mu_{e}}-\vec{\mu_{g}}\right)}^{2}}{hca^{3}}.$$

Here, $\vec{\mu_{e}}$ and $\vec{\mu_{g}}$ are vectors of electric dipole moments in the excited and ground state, respectively, *h* is the Plank’s constant, *c* is the velocity of light, *a* is the radius of the Onsager cavity. Solvent polarity function f(ε,n) depends on the electric permittivity ε and the refractive index *n* [27].

Due to the assumptions made about the shape of the cavity of the molecule (ellipsoidal) and the values of the polarizability of the molecule (α), including the values of the coefficient $2\alpha /a^{3}$, there are various models according to which the polarity functions f(ε,n) can be determined [27,28,29,30,31]. Therefore, the solvent polarity parameter, $f\left(\varepsilon ,n\right)$ can be calculated in various ways depending on the assumptions made.

(1) If the polarizability of the solute is neglect (α=0), the solvent polarity function obtained by Lippert and Mataga [28,29] can be calculated as:
  (5) $$f_{LM}\left(\varepsilon ,n\right)=\frac{\varepsilon -1}{2\varepsilon +1}-\frac{n^{2}-1}{2n^{2}+1}.$$
(2) According to McRae’s [30] theory:
  (6) $$f_{MR}\left(\varepsilon ,n\right)=\frac{\varepsilon -1}{\varepsilon +2}-\frac{n^{2}-1}{n^{2}+2}.$$
(3) According to theory proposed by Bakhshiev [31]:
  (7) $$f_{B}\left(\varepsilon ,n\right)\;=\;\frac{2n^{2}+1}{n^{2}+2}\left(\frac{\varepsilon -1}{\varepsilon +2}-\frac{n^{2}-1}{n^{2}+2}\right).$$
(4) According to Bilot–Kawski model [27], the polarity function takes the form:
  (8) $$f_{BK}\left(\varepsilon ,n\right)\;=\;\frac{\frac{\varepsilon -1}{2\varepsilon +1}-\frac{n^{2}-1}{2n^{2}+1}}{\left(1-\frac{2\alpha }{a^{3}}\frac{\varepsilon -1}{2\varepsilon +1}\right){\left(1-\frac{2\alpha }{a^{3}}\frac{n^{2}-1}{2n^{2}+1}\right)}^{2}}.$$

Kawski et al. have shown that, for an isotropic polarizability of the solute, the condition $2\alpha /a^{3}$ = 1 is frequently satisfied; thus, $f_{BK}\left(\varepsilon ,n,2\alpha /a^{3}=1\right)=f_{B}\left(\varepsilon ,n\right)$.

In order to find the value of the polarizability of the molecule, theoretical model (multiparametric linear regression analysis) proposed by Bayliss, McRae, and Ooshika [30,48,49] can be used. This model enables the determination of both α and Δμ values of the molecule under study. Shifts of the maxima of the absorption (*a*) and emission (*f*) spectra can by described by following relationships [51]:

(9) $$\Delta {\tilde{\nu }}_{a}={\tilde{\nu }}_{a}^{slolvent}-{\tilde{\nu }}_{a}^{gas}=Af\left(n\right)+Bf\left(\varepsilon ,n\right)+Cf{\left(\varepsilon ,n\right)}^{2},$$

(10) $$\Delta {\tilde{\nu }}_{f}={\tilde{\nu }}_{f}^{solvent}-{\tilde{\nu }}_{f}^{gas}=Af\left(n\right)+B_{1}f\left(\varepsilon ,n\right)+C_{1}f{\left(\varepsilon ,n\right)}^{2},$$

(11) $$\Delta {\tilde{\nu }}_{a}-\Delta {\tilde{\nu }}_{f}=\left(B-B_{1}\right)f\left(\varepsilon ,n\right)+\left(C-C_{1}\right)f{\left(\varepsilon ,n\right)}^{2},$$

where the functions can be calculated from: $f\left(n\right)=\left(n^{2}-1\right)/\left(2n^{2}+1\right)$ and $f\left(\varepsilon ,n\right)=\left(\varepsilon -1\right)/\left(2\varepsilon +1\right)-\left(n^{2}-1\right)/\left(2n^{2}+1\right)$. Differences $B-B_{1}$ and $C-C_{1}$ are given by:

(12) $$B-B_{1}=\frac{2}{hc}\frac{{\left(\mu_{e}-\mu_{g}\right)}^{2}}{a^{3}},$$

(13) $$C-C_{1}=\frac{2}{hc}\frac{\alpha_{e}-\alpha_{g}}{a^{6}}\left[3\mu_{e}^{2}-5\mu_{g}^{2}+2\mu_{e}\mu_{g}\right].$$

The *A* parameter corresponds to the contribution resulting from the nuclear polarizability of the solvent, while the *B* and $B_{1}$ parameters include the electron polarizability of the solvent [51].

#### 2.2.4. Theoretical Description of the Release of the Active Substance

The concentration of released active substance in the water solution was determined on the basis of the recorded absorption spectra. The drug concentration profiles consisted of two stages, corresponding to two mechanisms: diffusion (*D*) and relaxation (*R*). The concentration *C* (1/dm${}^{3}$) of drug released over time *t* from the polymer matrix can be described as follows:

(14) $$C\left(t\right)=C_{D}\left(t\leq t_{0}\right)+C_{R}\left(t\geq t_{0}\right),$$

where $C_{D}$ is concentration of released substance during first stage, and $C_{R}$ is concentration of released substance after time $t_{0}$, during second stage. Diffusion plays a main role in the initial phase of the release process. Later, in the second stage, the diffusion process is hampered by the polymer relaxation phenomenon.

The diffusion process of the release of the active substance from the matrix can be described by Korsmayer–Peppas equation [52,53]

(15) $$C_{D}\left(t\right)=k_{r}t^{n},$$

where $k_{r}$ is the rate of the release process and depends on the external conditions during the experiment, the glass transition temperature $T_{g}$ of polymer, and the hydrophilicity and structure of the matrix [54]. It is important to note that *n* indicates the dominant mechanism of the release process for cylindrical sample: 0.45 indicates Fickan diffusion (Case I), 0.85 is Case II (when domination of polymer stress relaxation process occurs), and 0.45 < *n* < 0.85 means anomalous diffusion (non-Fickan) [55,56].

The second stage in the release process is related to the polymer relaxation and the experimental results in this process can be expressed by the following equation (Hopfenberg model) [57,58]:

(16) $$C_{R}\left(t\right)=C_{\infty }\left[1-\frac{8}{\pi^{2}}\exp\left(-Ft\right)\right],$$

where *F* is a parameter proportional to the diffusion coefficient, and $C_{\infty }$ is the equilibrium concentration of the released substance [59].

## 3. Results and Discussion

### 3.1. Solvent Effect on the Absorption and Emission Spectra

The spectral characteristics of PAR, ACT, and NEA were registered in 14 solvents of different polarity. Solvents were selected taking into account their ability to non-specific and/or specific interactions with the studied molecules and ranked with increasing solvent polarity. Aprotic solvents (1–9) with low values of *SA* and *SB* parameters according to the Catalán solvent polarity scale (see description in Multiparametric Linear Regression Analysis section) and polar protic solvents (10–14) with high values of *SA* and *SB* parameters are collected in Table 1. The solvent parameters values: SP, SdP, SA, and SB were taken from literature data [45,46] and also collected in Table 1.

**Figure 2:**
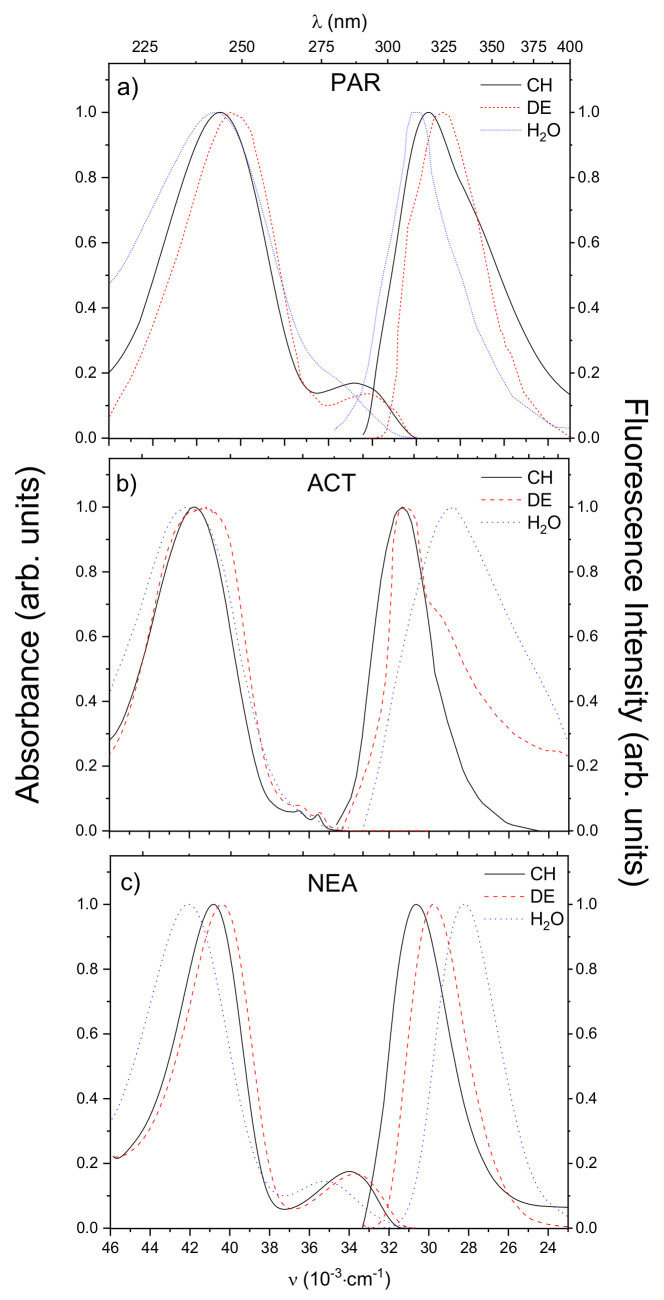
The long-wavelength absorption and fluorescence spectra of (**a**) PAR, (**b**) ACT and (**c**) NEA in solvents of different polarity: cyclohexane (CH, black straight line), diethyl ether (DE, red dashed line), and in deionized water (H${}_{2}$O, blue dotted line).

Figure 2 presents some representative absorption and fluorescence (obtained by exciting at the maximum of the absorption spectrum) spectra of the molecules under study in selected solvents of different polarity (non-polar cyclohexane (CH), medium polar, aprotic diethyl ether (DE), and strongly polar, protic deionized water (H${}_{2}$O). As can be seen the absorption spectra of PAR and its two analoges ACT and NEA in all studied solvents reveal two main bands: intensive, short-wavelength localized in range 230–260 nm and weak, broad, long-wavelength observed at 260–300 nm (see Figure 2). The values of extinction coefficients changes in the range 200–4000 M${}^{-1}\cdot$cm${}^{-1}$ for PAR, 1000–3000 M${}^{-1}\cdot$cm${}^{-1}$ for ACT and 1000–2000 M${}^{-1}\cdot$cm${}^{-1}$ for NEA (referring to spectra around 266 nm for all solvents). It is worth noting that only in the case of ACT the long-wavelength band shows pronounced vibrational structure, while, for the other two molecules (PAR, NEA), the vibrational structure is remarkably blurred. The fluorescence spectrum of all studied molecules in all chosen solvents possesses single, structureless and broad band localized in the spectral range 290–400 nm. It is also visible that the absorption and emission spectra of all compounds in non-polar CH (Figure 2) show an approximate mirror symmetry, suggesting that there is only a single excited electronic state contributing to the absorption and emission spectra.

**Figure 3:**
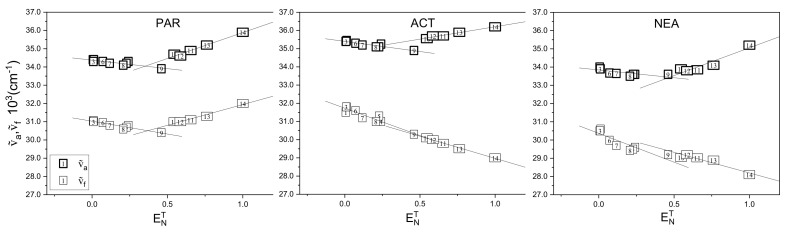
Positions of the long-wavelength absorption ($\tilde{\nu_{a}}$) and fluorescence ($\tilde{\nu_{f}}$) bands as a function of E${}_{N}^{T}$ parameter.

In Table 1 are also presented the long-wavelength absorption (a) and fluorescence (f) maxima positions (in cm${}^{-1}$) of the tested molecules in all used solvents. As can be seen in Table 1 and Figure 3, for all three compounds dissolved in aprotic solvents, upon increasing solvent polarity, maximum position of the long-wavelength absorption and emission band is red-shifted. This effect is more pronounced for the fluorescence spectra. It shows that compounds are more stabilized in the excited state rather than in the ground state.

Analyzing the data assembled in Figure 2 and Figure 3 and Table 1, one can state that the wavelength of maximum intensity of the long-wavelength absorption band of all molecules in polar protic solvents (10–14) is shifted towards shorter wavelengths (blue-shifted) as the polarity of the solvent increases, whereas an opposite behavior was observed for fluorescence band. The only exception is the spectrum of the PAR molecule. These effects imply the possibility of hydrogen bond interactions between the compounds and polar protic solvents. For all compounds dissolved in aprotic systems, the bathochromic shifts of the emission spectra with increasing solvent polarity are more pronounced than the shifts of the absorption spectra, which indicate that value of dipole moment increases upon excitation ($\mu_{e}>\mu_{g}$).

As it was mentioned earlier, the E${}_{N}^{T}$ scale is one of the most popular solvent polarity scales using to describe the solvent effect on the spectral behavior. Figure 3 presents a plot of the maxima of the absorption ($\tilde{\nu_{a}}$) and fluorescence ($\tilde{\nu_{f}}$) versus E${}_{N}^{T}$ parameter. As can be seen, solvent effects on $\tilde{\nu_{a}}$ and $\tilde{\nu_{f}}$ do not follow the classical behavior depending on E${}_{N}^{T}$ parameter, i.e., the spectroscopic data for aprotic solvents follow a roughly linear dependence on E${}_{N}^{T}$, while the protic media, forming a separate class, fall on another line. A double linear correlation indicates that, in protic solvents, specific interaction between solute and protic solvent molecule (hydrogen bonding) is occurring in addition to the well-known dipole-dipole interactions.

### 3.2. Multiparametric Linear Regression Analysis (MLR)

As it was mentioned, the positions of maxima and shape of the absorption and emission spectra reflect the nature of the solute–solvent interactions [60,61,62]. For this purpose, the multiple linear regression analysis proposed by Catalán was applied for each of the compounds dissolved in all 14 solvents. For all investigated systems, the position of the long-wavelength absorption ($\tilde{\nu_{a}}$) and fluorescence maxima ($\tilde{\nu_{f}}$) were analyzed applying Catalán multiparametric correlation using four-parameters scale (see Equation (2)). In the fitting procedure, the spectroscopic data listed in Table 1 were used. The obtained regression equations are presented in Equations (17)–(21). These equations show the relative contributions of each free fitting parameter, reflecting which interactions dominate in the studied system.

**PAR**:

(17) $$\begin{array}{c} \begin{array}{cc} \tilde{\nu_{a}}\;\left[{\mathrm{cm}}^{-1}\right] & =(34605\pm 588)-(388\pm 905)SP-(471\pm 124)SdP+(1909\pm 130)SA\\ & +\left(123\pm 120\right)SB,\;R^{2}=0.96, \end{array} \end{array}$$

relative contribution: SP–13%, SdP–16%, SA–66%, SB–4%

(18) $$\begin{array}{c} \begin{array}{cc} \tilde{\nu_{f}}\;\left[{\mathrm{cm}}^{-1}\right] & =(30912\pm 396)-(211\pm 609)SP-(669\pm 83)SdP+(1478\pm 88)SA\\ & +\left(1\pm 81\right)SB,\;R^{2}=0.96, \end{array} \end{array}$$

relative contribution: SP–9%, SdP–28%, SA–63%, SB–0%

**ACT**:

(19) $$\begin{array}{c} \begin{array}{cc} \tilde{\nu_{a}}\;\left[{\mathrm{cm}}^{-1}\right] & =(35880\pm 416)-(802\pm 639)SP-(593\pm 88)SdP+(1421\pm 92)SA\\ & +\left(272\pm 85\right)SB,\;R^{2}=0.96, \end{array} \end{array}$$

relative contribution: SP–26%, SdP–19%, SA–46%, SB–9%

(20) $$\begin{array}{c} \begin{array}{cc} \tilde{\nu_{f}}\;\left[{\mathrm{cm}}^{-1}\right] & =(31270\pm 121)+(812\pm 987)SP-(1202\pm 257)SdP-(1671\pm 270)SA\\ & -\left(285\pm 250\right)SB,\;R^{2}=0.94, \end{array} \end{array}$$

relative contribution: SP–20%, SdP–30%, SA–42%, SB–7%

**NEA**:

(21) $$\begin{array}{c} \begin{array}{cc} \tilde{\nu_{a}}\;\left[{\mathrm{cm}}^{-1}\right] & =(33358\pm 570)+(962\pm 878)SP-(295\pm 120)SdP+(1329\pm 126)SA\\ & -\left(477\pm 117\right)SB,\;R^{2}=0.94, \end{array} \end{array}$$

relative contribution: SP–31%, SdP–10%, SA–43%, SB–16%

(22) $$\begin{array}{c} \begin{array}{cc} \tilde{\nu_{f}}\;\left[{\mathrm{cm}}^{-1}\right] & =(31732\pm 656)-(1934\pm 1013)SP-(1248\pm 139)SdP-(884\pm 146)SA\\ & -\left(258\pm 135\right)SB,\;R^{2}=0.97, \end{array} \end{array}$$

relative contribution: SP–45%, SdP–29%, SA–20%, SB–6%.

**Figure 4:**
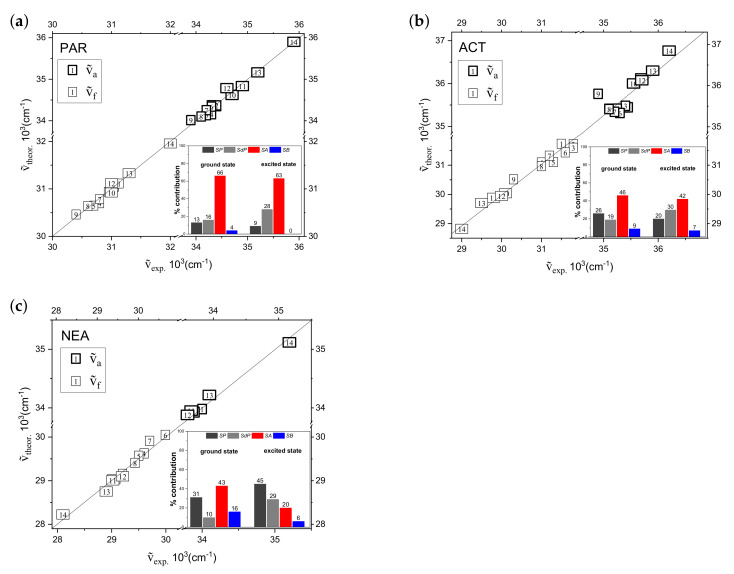
Position of the long-wavelength absorption and fluorescence bands of (**a**) PAR, (**b**) ACT, and (**c**) NEA obtained according to Catalán model ( ${\tilde{\nu }}_{a,f}^{\mathrm{theor}}$) versus the corresponding experimental $\tilde{\nu_{a}}$ and $\tilde{\nu_{f}}$ values. The relative (percentage) contribution of non-specific and specific interactions is presented in the inset of Figure 4.

In order to better visualize the goodness of the fitting procedure, Figure 4 presents the absorption and fluorescence maxima of molecules under study calculated according to Equations (17)–(21) versus the corresponding experimental $\tilde{\nu_{a}}$ and $\tilde{\nu_{f}}$ values. The correlation coefficient (0.94 $\leq R^{2}\leq$ 0.97) indicates a good quality of the fitting procedure. Additionally, to facilitate the analysis of the nature of the solute-solvent interactions, the relative (percentage) contributions of non-specific and specific interactions on PAR, ACT, and NEA solvatochromism are also presented in the insets in Figure 4.

From the analysis of $\tilde{\nu_{a}}$ and $\tilde{\nu_{f}}$ of PAR molecule according to Catalán equation it is clear that the specific interactions between PAR and protic solvents are present either in its ground and excited states. In this case, two parameters, solvent acidity (SA) (mainly) and solvent polarizability/dipolarity (SP+SdP) play a crucial role. The position of absorption (*a*) and fluorescence (*f*) band maximum is determined in S${}_{SA,a}$ = 66% and S${}_{SA,f}$ = 63% by solvent acidity and in (S${}_{SP}$ + S${}_{SdP}$)${}_{a}$ = 29% and (S${}_{SP}$ + S${}_{SdP}$)${}_{f}$ = 37% by dipolar interactions. These results also suggest that the influence of the solvent basicity (SB parameter) on $\tilde{\nu_{a}}$ and $\tilde{\nu_{f}}$ values may be considered as negligible (S${}_{SB,a}$ = 4% and S${}_{SB,f}$ = 0%). This finding indicates the involvement of the only hydroxyl group (-OH) in specific interactions between PAR and protic molecules.

Multiple-linear correlation analysis shows, in general, that non-specific dipole-dipole interactions (SP+SdP) and solvent acidity (SA) have also major influence on absorption and emission solvatochromism of ACT and NEA, whereas solvent basicity has very minor influence. Moreover, for both molecules (ACT and NEA), polarity parameters (S${}_{SP}$ + S${}_{SdP}$) contribute more in the excited state than ground state ((S${}_{SP}$ + S${}_{SdP}$)${}_{a}$(ACT) = 35% versus (S${}_{SP}$ + S${}_{SdP}$)${}_{f}$(ACT) = 50% and (S${}_{SP}$ + S${}_{SdP}$)${}_{a}$(NEA) = 41% versus (S${}_{SP}$ + S${}_{SdP}$)${}_{f}$(NEA) = 74%), indicating that the value of electric dipole moment increases upon excitation ($\mu_{e}>\mu_{g}$). It is also important to note that, for both PAR analoges, the solvation process is more determined (in comparison with PAR) by dipolar interactions, i.e., (S${}_{SP}$ + S${}_{SdP}$)${}_{a}$(ACT) = 35% versus (S${}_{SP}$ + S${}_{SdP}$)${}_{a}$(PAR) = 29% and (S${}_{SP}$ + S${}_{SdP}$)${}_{f}$(ACT) = 50% versus (S${}_{SP}$ + S${}_{SdP}$)${}_{f}$(PAR) = 37% and (S${}_{SP}$ + S${}_{SdP}$)${}_{a}$(NEA) = 41% versus (S${}_{SP}$ + S${}_{SdP}$)${}_{a}$(PAR) = 29% and (S${}_{SP}$ + S${}_{SdP}$)${}_{f}$(NEA) = 74% versus (S${}_{SP}$ + S${}_{SdP}$)${}_{f}$(PAR) = 37%. This behavior is understandable in terms of the chemical structures of the studied molecules, i.e., differently substituted analoges.

### 3.3. Determination of the Changes in the Dipole Moment

As it was mentioned, the solute-solvent interactions can be also described in terms of the changes in the dipole moment of fluorophore upon excitation. In order to obtain more insight about electronic charge distribution of the investigated molecules, in the ground and excited states, the correlations of the Stokes shift with different polarity functions were analyzed according to the theoretical models of solvatochromism proposed by McRae, Bakhshiev, Lippert, and Mataga.

**Figure 5:**
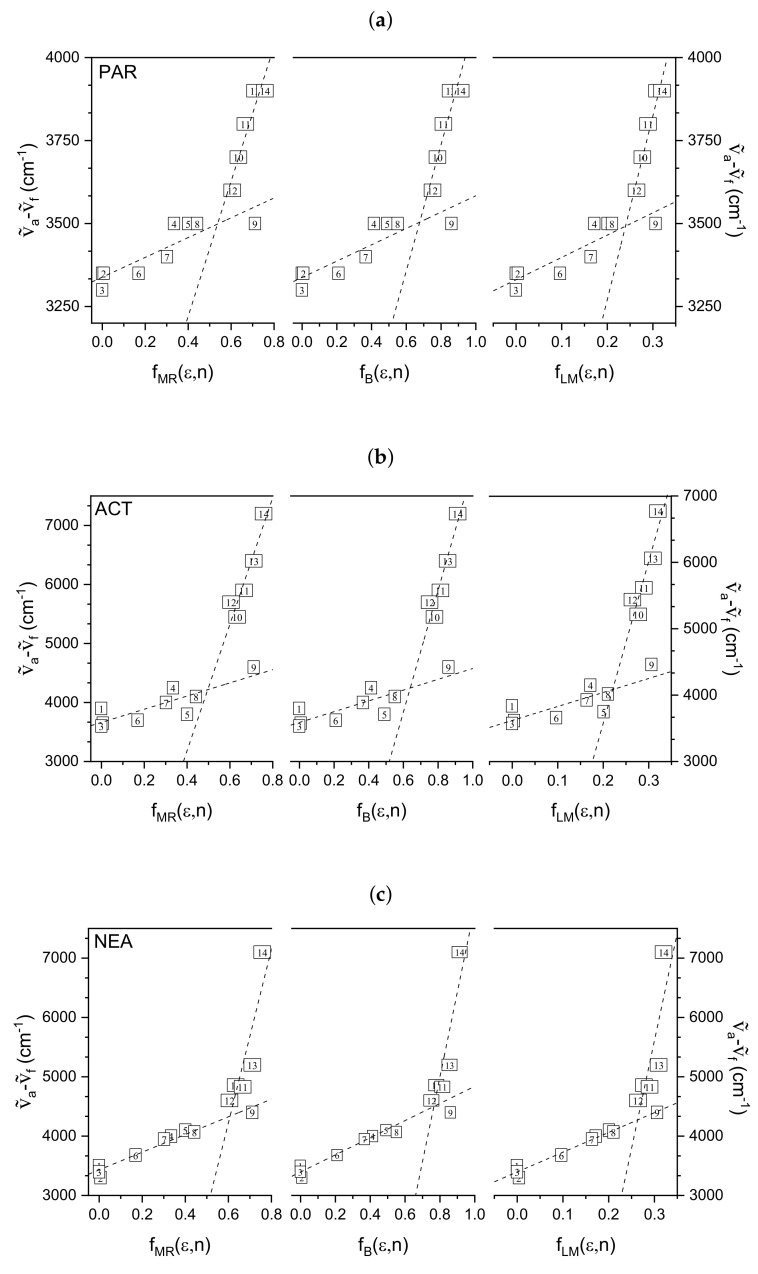
Stokes shifts ($\tilde{\nu_{a}}-\tilde{\nu_{f}}$) versus the solvent polarity functions ($f_{MR}\left(\varepsilon ,n\right)$, $f_{B}\left(\varepsilon ,n\right)$, $f_{LM}\left(\varepsilon ,n\right)$) for three tested molecules: (**a**) paracetamol (PAR), (**b**) acetanilide (ACT), and (**c**) *N*-ethylaniline (NEA).

Figure 5 presents the Stokes shifts ($\tilde{\nu_{a}}-\tilde{\nu_{f}}$) versus the solvent polarity functions ($f_{MR}\left(\varepsilon ,n\right)$, $f_{B}\left(\varepsilon ,n\right)$, $f_{LM}\left(\varepsilon ,n\right)$) for three tested molecules. It is easy to see that, for all investigated systems, the data presented do not fit well to the one, linear function defined by Equation (3). There is an adequate linear relationship between $\tilde{\nu_{a}}-\tilde{\nu_{f}}$ and analyzed polarity functions for two different groups of solvents (protic and aprotic). This behavior confirms the existence of hydrogen bond formed between the solute molecule and protic solvents. Taking into account above the difference in the ground and excited state dipole moments were determined for free molecules (PAR, ACT, NEA) and hydrogen-bonded complexes (investigated compound+protic solvent).

To calculate the excited state dipole moment ($\mu_{e}$), the ground state dipole moment ($\mu_{g}$) and the Onsager’s cavity radius (*a*) are necessary. For all molecules, they were obtained from the theoretical calculations using CAChe WS 5.04 computer program. The results are assembled in Table 2.

The values of $m_{1}$ determined separately for aprotic (1–9) and protic (10–14) solvents, correlation coefficients and difference in the ground and excited state dipole moment (Δμ) obtained on the basis of McRae (MR), Bakhshiev (B), and Lippert and Mataga (LM) models are also summarized in Table 2.

It should be noticed that Δμ values determined using three different models of solvation for protic solvents (which are capable of forming hydrogen bonds with investigated molecules) differ significantly from those determined for aprotic sovents. Based on the data from Table 2 it is clear that Δμ for hydrogen-bonded complexes is ca. 2.7, 3.3, and 3.2 times higher than that for free PAR, ACT, and NEA molecules, respectively. This behavior indicates that specific, hydrogen-bonding interactions cause the strong redistribution of charge in the excited state.

It is also evident that Δμ values determined by three solvatochromic models differ from each other. For all investigated systems, the lowest values were obtained based on Bakhshiev’s model, whereas the highest ones for Lippert-Mataga model. Moreover, $\Delta \mu_{B}$ and $\Delta \mu_{MR}$ values are nearly the same $\Delta \mu_{B}\approx \Delta \mu_{MR}=\langle \Delta \mu \rangle$ (differ only by about 5%), whereas significant difference exists between $\Delta \mu_{B}\approx \Delta \mu_{MR}$ and $\Delta \mu_{LM}$. The noted difference between $\Delta \mu_{B}\approx \Delta \mu_{MR}$ and $\Delta \mu_{LM}$ is understandable in terms of the solute polarizability effect. As it was mentioned earlier, the Lippert-Mataga model assumes that the polarizability of the solute is negligible, while Bakhshiev’s and McRae’s models assume that polarizability factor $2\alpha /a^{3}$ = 1. Thus, significant differences in Δμ values determined using discussed theoretical models clearly indicate that the solute polarizability affects the change in dipole moment of the investigated systems upon excitation.

Taking into account above the effect of the solute polarizability on the Δμ values was analyzed according to the Bilot-Kawski approach for different $2\alpha /a^{3}$ parameters (0 ≤ $2\alpha /a^{3}$ ≤ 1). The determined difference in the ground and excited state dipole moments values of all molecules dissolved in aprotic systems obtained for different $f_{BK}\left(\varepsilon ,n,0\leq 2\alpha /a^{3}\leq 1\right)$ parameters are collected in Table 3. It is seen that $\Delta \mu_{BK}$ values decrease continuously with increasing $2\alpha /a^{3}$ parameter. The $\Delta \mu_{BK}$ values determined for two final values of $2\alpha /a^{3}$, i.e., $2\alpha /a^{3}$ = 0 and 1, differ by about 20% for all molecules, which confirms that solute polarizability affects the ground and excited state dipole moments.

Taking into account that: (**i**) for all molecules, the experimental error in determination of ${\tilde{\nu }}_{0(a)}$ and ${\tilde{\nu }}_{0(f)}$ using Catalàn models equals about 500 cm${}^{-1}$, (**ii**) for all investigated compounds dissolved in protic solvents, both free molecules and hydrogen-bonded complex occurs, which means that the designed ${\tilde{\nu }}_{0(a)}$ and ${\tilde{\nu }}_{0(f)}$ values should be interpreted as average ${\tilde{\nu }}_{0}$ values of two existing species (unbond (free) molecule and hydrogen-bonded complex), (**iii**) the values of Δμ and $2\alpha /a^{3}$ calculated using Equations (9)–(13) are very sensitive to the choice of ${\tilde{\nu }}_{0(a)}$ and ${\tilde{\nu }}_{0(f)}$, transition energies in the gas phase were calculated as mean values of two alternatively determined values $\left(\left({\tilde{\nu }}_{0}^{\mathrm{apr}.}\left(C\right)+{\tilde{\nu }}_{0}^{E_{N}^{T}}\right)/2\right)$. The ${\tilde{\nu }}_{0}^{\mathrm{apr}.}\left(C\right)$ corresponds to the transition energy in the gas phase obtained on the basis of the Catalàn model applied for aprotic (apr.) systems and ${\tilde{\nu }}_{0}^{E_{N}^{T}}$ corresponds to the gas phase transition energy calculated for linear relationship between ${\tilde{\nu }}_{a}^{\max}$(${\tilde{\nu }}_{f}^{\max}$) and E${}_{N}^{T}$ scale for aprotic solvents. Thus, the values obtained and Δμ and $2\alpha /a^{3}$ values obtained using multiple linear regression analysis are presented in Table 4.

Differences in the ground and excited states dipole moments for PAR, ACT, and NEA were determined to be 0.90 D, 4.07 D, and 2.95 D, whereas $2\alpha /a^{3}$ equal 1.0, 0.15, and 0.02. It is clearly seen that, for PAR and NEA, physical parameters Δμ and $2\alpha /a^{3}$ determined using regression analysis are almost the same (with the limit of experimental error) as Δμ values obtained using Bilot-Kawski method for $2\alpha /a^{3}$ = 1.0 (PAR) and 0.0 (NEA), whereas, for ACT, the Δμ value determined from multiparameter regression analysis differs from this calculated from Bilot-Kawski model. Summarizing, the obtained data for PAR indicate that solute polarizability has important impact on dipole moment values, while, for ACT and NEA, polarizability plays a less significant role.

### 3.4. Temperature-Dependent Release of Active Substances

As it was mentioned, the PAR, ACT, and NEA solvatochromic studies were conducted to investigate the nature of the solute–solvent interactions in liquid systems. Moreover, the obtained results can greatly facilitate the interpretation of the interactions (non-specific and specific) between the compounds under study and hydrated hydrogel matrix in terms of releasing and absorbing the drug. Taking into account that the studied molecules possess different values of dipole moments in the ground and excited states, as well as different hydrogen bond donor/acceptor ability, the studies of the release of PAR, ACT, and NEA from hydrogel matrices were carried out in order to determine the role of the chemical structure of the active substance (different substituents) on the kinetics of the release process.

**Figure 6:**
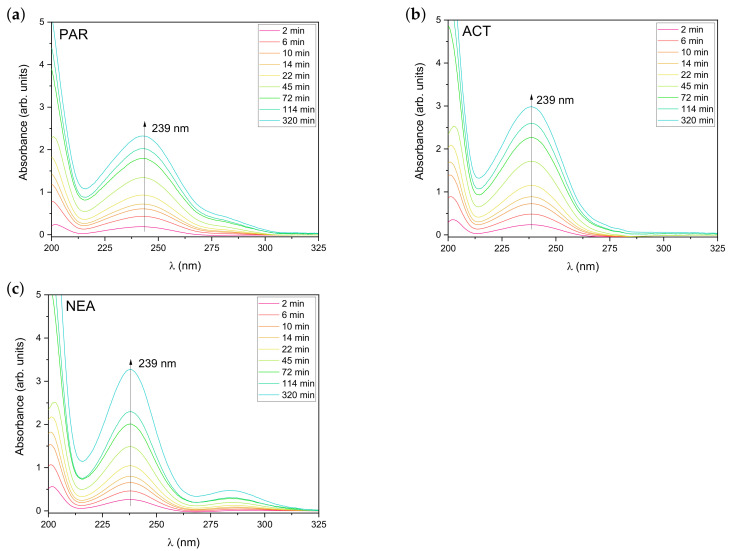
Changes in absorption spectra of (**a**) PAR, (**b**) ACT, and (**c**) NEA during release from PU/polyethylene glycol (PEG) 4000 hydrogel at room temperature (25 °C).

The steady-state absorption spectra of PAR, ACT, and NEA during release from hydrogel matrix (recorded as a function of the release time) are presented in Figure 6.

**Figure 7:**
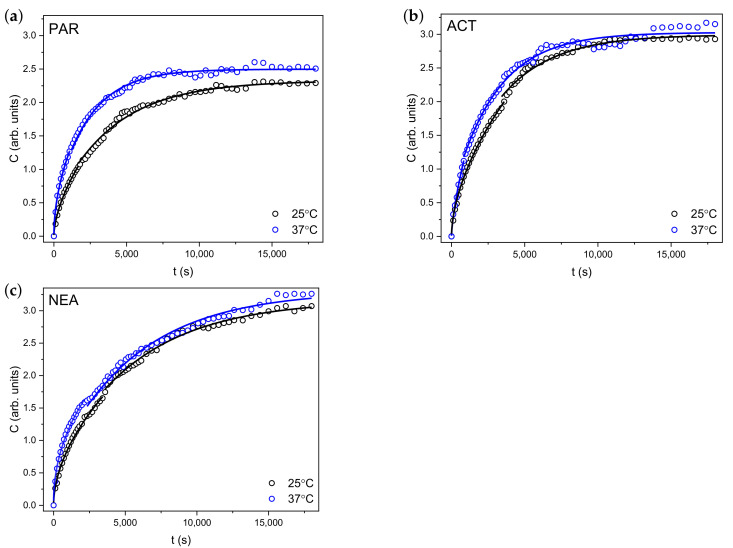
Changes in molar concentration (C) of (**a**) PAR, (**b**) ACT, and (**c**) NEA released from PU/PEG hydrogels at 25 °C (black circles) and at 37 °C (blue circles). The solid lines represent the fitted theoretical models.

It is clear from Figure 6 that, for all substances, the molar absorption coefficients of the long-wavelength absorption band with a maximum around 240 nm gradually increases with the release time. It can also be seen that, after 320 min (blue line), the more significant changes in absorption spectra were observed for NEA compared to PAR and ACT. In order to characterize the kinetics of the release process, the changes of the value of absorbance at 239 nm were analyzed. In order to better quantitatively visualize the release process and taking into account the fact that absorbance is directly proportional to the concentration of the released substance (*C*), Figure 7 shows the temporal evolution of the changes in solute concentration released from polyurethane hydrogel at room temperature (25 °C) and at 37 °C. As it is shown in Figure 7, the release curves clearly show the temperature dependence. It is important to note that changes in the concentration of the released substance are significantly larger in the first phase. As expected, for all molecules, release process (temporal evolution of C(t)) is significantly faster at higher temperature (37 °C). It can also be seen that the highest increase in the concentration of the released substance in the first phase at 37 °C was observed for PAR.

The C(t) plot is initially almost linear and then tends to approach a well-defined limit (plateau). This separation into two stages is due to the existence of two physical processes: diffusion (described by Equation (15)) and relaxation (Equation (16)).

It is assumed that the diffusion process accounts for about 60% of the amount of released substance [52]. In the case of our measurements, it turned out that this process affects only about 30–40% of the released substance [63,64]. It was difficult to separate the experimental points describing the diffusion process from the relaxation process around the transition from one process to the another. The selection of measurement points was made by controlling the accuracy of the fit to the theoretical formulas. The results are shown as solid lines in Figure 7. The parameters obtained as a result of the fitting are presented in Table 5.

Examining the $k_{r}$ values for PAR, ACT, and NEA at room temperature (25 °C), it is evident that the first stage is the fastest process in the case of ACT (2.18 × 10${}^{-2}$ s${}^{-n}$), whereas, in the case of the PAR (1.30 × 10${}^{-2}$ s${}^{-n}$) discussed, the process is significantly slower with increasing temperature. Moreover, the value of $k_{r}$ for PAR and NEA increases about two times, whereas, for the ACT, the value of $k_{r}$ presents opposite behavior.

The obtained *n* values at room temperature are in the 0.47÷0.69 range, which indicates that anomalous diffusion is involved in this process for all investigated samples. This means that the investigated process is controlled by both mechanisms (diffusion and relaxation). At 37 °C, the value of *n* decreases in the case of PAR and NEA, while, in the case of ACT, the factor *n* increases from 0.57 to 0.69, which indicates a greater role of relaxation than diffusion process in the transport of ACT. For all tested compounds, an increase in the equilibrium concentration of the released substance ($C_{\infty }$) at higher temperature is observed.

The *F* parameter, proportional to the diffusion coefficient, characterizing the relaxation process, has the lowest value for NEA. For this substance, the *F* value is practically independent of the temperature. The greatest influence of temperature on the value of *F* is observed in the case of PAR.

### 3.5. Analysis of Release Kinetics Parameters Based on the Results of Solvatochromic Analysis

From spectroscopic studies in liquid systems, it was obtained that solvation of the investigated molecules is mainly determined by non-specific, dipolar interactions, whereas specific (hydrogen bonds) interactions play a less significant role. This suggests that PAR and its two analoges trapped in polymer network and gradually released from the hydrogel into water, can interact with matrix both in specific (hydrogen bonding) and non-specific (dipolar) way.

**Figure 8:**
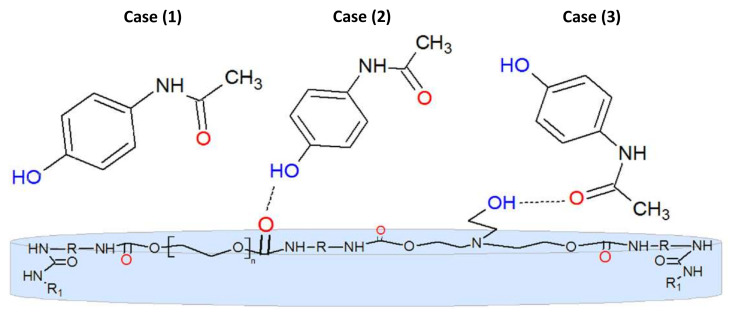
Three possible interactions of a PAR molecule with a hydrogel matrix.

Taking into account the polymer chain structure and chemical structure of the investigated molecules, as well as results of solvatochromic studies, we propose the three ways model of interactions of the studied molecules with matrix (Figure 8). Presented in Figure 8, the model assumes that, in addition to the usual non-specific (dipole-dipole) interactions (Case (1)), specific interactions between PAR and the components of the hydrogel matrix (Cases (2) and (3)) may occur.

As the PAR molecule contains two main groups capable of participating in specific interactions with matrix (i.e, hydrogen bond donation ability of the hydroxyl group (–OH) and hydrogen bond acceptor ability of the oxygen atom of the carbonyl group), Figure 8 presents the possible specific interactions (case (2) and case (3)).

Analyzing the chemical structure of ACT (Figure 1), it can be stated that only the carbonyl group (C=O) in this compound is the likely site for intermolecular hydrogen bondings in hydrogel matrix in the water environment (case (3) in Figure 8). Solvatochromic studies also confirms that this molecule is incapable of hydrogen-bond donor abilities. Finally, it should by recalled that chemical structure of NEA molecule (Figure 1) practically excludes the formation of a hydrogen bonds with the matrix.

Examining the $k_{r}$ values obtained for each of the fluorophores at room temperature, it is evident that $k_{r}$ for PAR molecule is lower than for ACT and NEA. On the other hand, $k_{r}$ values for ACT and NEA are essentially the same with the limit of experimental error. This observations indicate that (**i**) specific interactions between PAR and matrix in the water environment are not negligible, hindering its release, (**ii**) release process of ACT and NEA is mainly determined by dipolar interactions, whereas the specific interactions in the case of ACT play an insignificant role, and (**iii**) only one type from hydrogen bonds is important in the release process of PAR, namely the one related to the hydroxyl hydrogen.

The multiparametric linear regression analysis (see Equations (17)–(21)) applied for all fluorophores dissolved in liquid environments shows that the contribution of the SB parameter, representing the solvent’s hydrogen bond acceptor strength, is negligible. On the other hand, quantitative analysis of the release kinetics clearly indicates that hydroxyl group (–OH) in PAR molecule is the likely site for intermolecular hydrogen bondings in hydrogel matrix in the water environment. This result is understandable in terms of synthesis conditions, i.e., during the synthesis of the matrix, care was taken that the number of diisocyanate groups in relation to the cross-linking agent (triethanolamine) was predominant. This leads to cross-linking of the matrix mainly through hydroxyl groups (the absence of unbound –OH groups), which means that the situation (3) in Figure 8 seems very unlikely. Thus, delay in release of PAR from hydrogel matrix compared to ACT and NEA can, therefore, be explained by the presence of specific hydrogen bonds, i.e., PAR interacts with the matrix mainly through the hydroxyl group –OH (case (2)), which does not occur in the case of the other molecules.

As expected, the temperature increase results also in weakness of the above described interactions between PAR and matrix, which is confirmed by the larger difference between the $k_{r}$ values at both temperatures (see Table 5).

## 4. Conclusions

The main goal of these combined solvatochromic investigations in liquid system and drug release studies from hydrogel matrix was to reveal substituent effect and its influence on the nature of the solute–solvent and solute–hydrogel matrix interactions. Performed studies clearly show that all compounds under studies (in the ground and excited states) in protic media form a solute–solvent hydrogen–bonded complex. Moreover, our results suggest that spectroscopic characteristics (i.e., absorption and emission spectra) are mainly determined by solvent acidity and solvent polarity/polarizability, whereas the influence of the solvent basicity on ${\tilde{\nu }}_{a}$ and ${\tilde{\nu }}_{f}$ values may be considered as negligible. From the spectroscopic studies in liquid systems, it is evident that the solute polarizability strongly affects the ground and excited state dipole moments of PAR molecule.

It is important to note that we observed that specific drug–hydrogel matrix hydrogen bonding interactions provide stabilization by reducing the drug mobility. Quantitative analysis of the release kinetics indicates that hydroxyl group of PAR is the likely site for intermolecular hydrogen bondings in hydrogel matrix.

## Figures and Tables

**Table 1:** Solvent parameters (E${}_{N}^{T}$ and Catalán parameters) applied in the calculations, values of the solvent polarity functions ($f_{MR}\left(\varepsilon ,n\right)$,$f_{B}\left(\varepsilon ,n\right)$,$f_{LM}\left(\varepsilon ,n\right)$) and the position of the long-wavelength absorption ($\tilde{\nu_{a}}$) and fluorescence ($\tilde{\nu_{f}}$) maxima of PAR, ACT, and NEA.

Solvent *	$E_{N}^{T}$	Catalán Parameters				$f_{\mathrm{MR}}\left(\varepsilon ,n\right)$	$f_{B}\left(\varepsilon ,n\right)$	$f_{\mathrm{LM}}\left(\varepsilon ,n\right)$	$\tilde{\nu_{a}}$ (cm${}^{-1}$)			$\tilde{\nu_{f}}$ (cm${}^{-1}$)		
		*SP*	*SdP*	*SA*	*SB*				PAR	ACT	NEA	PAR	ACT	NEA
1 CH	0.006	0.683	0	0	0.073	−0.00118	−0.00148	−0.00075	34,400	35,400	34,000	31,050	31,500	30,500
2 2,2,4-TMP	0.012	0.618	0	0	0.044	0.00591	0.00731	0.00385	34,400	35,450	33,900	31,050	31,800	30,600
3 HX	0.009	0.616	0	0	0.056	−0.00064	−0.00078	−0.00042	34,300	35,400	33,900	31,000	31,800	30,500
4 BA	0.241	0.674	0.535	0	0.525	0.33402	0.41352	0.17163	34,300	35,250	33,600	30,800	31,000	29,600
5 EA	0.228	0.656	0.603	0	0.542	0.39986	0.49026	0.20058	34,200	35,100	33,600	30,700	31,300	29,500
6 BE	0.071	0.672	0.175	0	0.637	0.16874	0.20936	0.09649	34,300	35,300	33,670	30,950	31,600	29,990
7 DE	0.117	0.617	0.385	0	0.562	0.30123	0.36597	0.16354	34,200	35,200	33,650	30,800	31,200	29,700
8 THF	0.207	0.714	0.634	0	0.591	0.44175	0.55002	0.21033	34,100	35,100	33,500	30,600	31,000	29,430
9 AcN	0.460	0.645	0.974	0.044	0.286	0.71076	0.86014	0.30575	33,900	34,900	33,600	30,400	30,300	29,200
10 2-PrOH	0.546	0.633	0.808	0.283	0.830	0.63411	0.77934	0.27692	34,700	35,550	33,900	31,000	30,100	29,040
11 EtOH	0.654	0.633	0.783	0.400	0.658	0.66664	0.81354	0.28948	34,900	35,700	33,850	31,100	29,800	29,020
12 BuOH	0.586	0.674	0.655	0.341	0.809	0.60520	0.75107	0.26414	34,600	35,700	33,800	31,000	30,000	29,200
13 MtOH	0.762	0.608	0.904	0.605	0.545	0.71140	0.85514	0.30930	35,200	35,900	34,100	31,300	29,500	28,900
14 H${}_{2}$O	1.000	0.681	0.997	1.062	0.025	0.75725	0.91280	0.32008	35,900	36,200	35,200	32,000	29,000	28,100

* CH–cyclohexane, 2,2,4-TMP–2,2,4-trimethylpentane, HX–hexane, BA–buthyl acetate, EA–ethyl acetate, BE–buthyl ether, DE–diethyl ether, THF–tetrahydrofuran, AcN–acetonitrile, 2-PrOH–2-propanol, EtOH–ethanol, BuOH–buthanol, MtOH–methanol, H${}_{2}$O–deionized water.

**Table 2:** The theoretically calculated ground state dipole moments ($\mu_{g}$ (D)) and Onsager’s radius values (*a* (Å)), as well as the values of $m_{1}$, difference in the excited and ground state dipole moments Δμ (D), and excited state dipole moment $\mu_{e}$ (D) of the investigated molecules obtained using McRae’s, Bakhshiev’s, and Lippert-Mataga’s models.

	Solvent Number	PAR			ACT			NEA		
		$\mu_{g}$ = 2.61 (D)		*a* = 3.03 (Å)	$\mu_{g}$ = 3.32 (D)		*a* = 3.02 (Å)	$\mu_{g}$ = 1.64 (D)		*a* = 3.05 (Å)
		$m_{1}$(cm${}^{-1}$)	Δμ (D)	$\mu_{e}$ (D)	$m_{1}$(cm${}^{-1}$)	Δμ (D)	$\mu_{e}$ (D)	$m_{1}$(cm${}^{-1}$)	Δμ (D)	$\mu_{e}$ (D)
**McRae**	1–9	298	0.91	3.52	1116	1.74	5.06	1489	2.05	3.69
	10–14	2034	2.37	4.98	10717	5.40	8.72	14,636	6.43	8.07
**Bakhshiev**	1–9	246	0.82	3.43	914	1.58	4.90	1444	2.02	3.66
	10–14	1899	2.29	4.90	1032	5.30	8.62	14,295	6.35	7.99
**Lippert-Mataga**	1–9	672	1.36	3.97	2372	2.54	5.86	3330	3.04	4.69
	10–14	5538	3.91	6.52	27425	8.65	12.00	36,296	10.12	11.80

**Table 3:** Polarizability-dependent polarity function $f_{BK}\left(\varepsilon ,n,0\leq 2\alpha /a^{3}\leq 1\right)$, values of $\Delta \mu_{BK}$ (D) and $\mu_{e,BK}$ (D) for PAR, ACT, and NEA as a function of $2\alpha /a^{3}$ parameter.

Solvent	$f_{\mathrm{BK}}\left(\varepsilon ,n,0\leq 2\alpha /a^{3}\leq 1\right)$										
	0.0	0.1	0.2	0.3	0.4	0.5	0.6	0.7	0.8	0.9	1.0
1 CH	−0.00075	−0.00080	−0.00085	−0.00090	−0.00096	−0.00103	−0.00110	−0.00118	−0.00127	−0.00137	−0.00148
2 2,2,4−TMP	0.00385	0.00408	0.00433	0.00460	0.00490	0.00522	0.00556	0.00594	0.00636	0.00681	0.00731
3 HX	−0.00042	−0.00045	−0.00047	−0.00050	−0.00053	−0.00057	−0.00060	−0.00064	−0.00069	−0.00073	−0.00078
4 BA	0.17163	0.18516	0.20020	0.21696	0.23572	0.25679	0.28056	0.30747	0.33811	0.37317	0.41352
5 EA	0.20058	0.21652	0.23427	0.25410	0.27635	0.30140	0.32975	0.36198	0.39883	0.44120	0.49026
6 BE	0.09649	0.10335	0.11088	0.11918	0.12833	0.13845	0.14968	0.16219	0.17614	0.19178	0.20936
7 DE	0.16354	0.17546	0.18860	0.20313	0.21925	0.23719	0.25722	0.27968	0.30495	0.33352	0.36597
8 THF	0.21033	0.22815	0.24813	0.27061	0.29603	0.32490	0.35787	0.39572	0.43945	0.49034	0.55002
9 AcN	0.30575	0.33260	0.36295	0.39745	0.43688	0.48227	0.53487	0.59637	0.66894	0.75551	0.86014
**Molecule**	$\Delta \mu_{BK}$ (D) as a function of $2\alpha /a^{3}$										
PAR	1.36	1.31	1.25	1.20	1.15	1.09	1.04	0.99	0.93	0.88	0.83
ACT	2.56	2.46	2.36	2.27	2.17	2.07	1.98	1.88	1.78	1.69	1.59
NEA	3.04	2.91	2.79	2.67	2.55	2.43	2.31	2.20	2.08	1.96	1.84
**Molecule**	$\mu_{e,BK}$ (D) as a function of $2\alpha /a^{3}$										
PAR	4.39	4.34	4.28	4.23	4.18	4.12	4.07	4.02	3.96	3.91	3.86
ACT	5.58	5.48	5.38	5.29	5.19	5.09	5.00	4.90	4.80	4.71	4.61
NEA	6.09	5.96	5.84	5.72	5.60	5.48	5.36	5.25	5.13	5.01	4.89

**Table 4:** Transition energies in the gas phase obtained on the basis of (**i**) Catalàn model for aprotic (apr.) systems $\left({\tilde{\nu }}_{0(a)}^{\mathrm{apr}.}\left(C\right),{\tilde{\nu }}_{0(f)}^{\mathrm{apr}.}\left(C\right)\right)$, (**ii**) linear relationship between ${\tilde{\nu }}_{a}^{\max}$(${\tilde{\nu }}_{f}^{\max}$) and E${}_{N}^{T}$$\left({\tilde{\nu }}_{0(a)}^{E_{N}^{T}},{\tilde{\nu }}_{0(f)}^{E_{N}^{T}}\right)$, and average of two alternatively determined values $\langle {\tilde{\nu }}_{0,(a)}^{\mathrm{apr}.}\rangle$ and $\langle {\tilde{\nu }}_{0,(f)}^{\mathrm{apr}.}\rangle$, as well as Δμ (D) and $2\alpha /a^{3}$ values obtained using multiple linear regression analysis.

Molecule	${\tilde{\nu }}_{0(a)}^{\mathrm{apr}.}\left(C\right)$	${\tilde{\nu }}_{0(f)}^{\mathrm{apr}.}\left(C\right)$	${\tilde{\nu }}_{0(a)}^{E_{N}^{T}}$	${\tilde{\nu }}_{0(f)}^{E_{N}^{T}}$	${\langle {\tilde{\nu }}_{0,(a)}^{\mathrm{apr}.}\rangle }^{*}$	${\langle {\tilde{\nu }}_{0,(f)}^{\mathrm{apr}.}\rangle }^{*}$	Δμ	$2\alpha /a^{3}$
PAR	34,224	31,086	34,032	31,027	34,128	31,057	0.90	1.00
ACT	35,399	32,876	35,400	31,728	35,400	32,302	4.07	0.15
NEA	33,802	30,440	33,841	30,364	33,822	30,402	2.95	0.02

* $\langle {\tilde{\nu }}_{0,(a,f)}^{\mathrm{apr}.}\rangle$ = $\left(({\tilde{\nu }}_{0(a,f)}^{\mathrm{apr}.}\left(C\right)+{\tilde{\nu }}_{0(a,f)}^{E_{N}^{T}})/2\right)$.

**Table 5:** The values of parameters describing the release process for studied hydrogel systems obtained by fitting Equations (15) and (16) (Korsmeyer–Peppas and Hopfenberg models) to two stages of release.

	PAR		ACT		NEA	
	25 ${}^{\circ }$C	37 ${}^{\circ }$C	25 ${}^{\circ }$C	37 ${}^{\circ }$C	25 ${}^{\circ }$C	37 ${}^{\circ }$C
$k_{r}$ (10${}^{-2}\cdot s^{-n}$)	1.30 ± 0.16	3.52 ± 0.42	2.18 ± 0.10	1.02 ± 0.22	1.91 ± 0.17	4.87 ± 0.40
*n*	0.59 ± 0.01	0.51 ± 0.02	0.57 ± 0.01	0.69 ± 0.04	0.53 ± 0.01	0.47 ± 0.01
$C_{\infty }$ (10${}^{-5}\cdot$ 1/dm${}^{3}$)	2.33 ± 0.01	2.50 ± 0.01	2.99 ± 0.01	3.03 ± 0.02	3.16 ± 0.02	3.31 ± 0.03
*F* (10${}^{-4}\cdot s^{-1}$)	2.50 ± 0.01	4.28 ± 0.01	2.84 ± 0.06	3.38 ± 0.08	1.74 ± 0.04	1.76 ± 0.05

## Data Availability

The data is provided by Marta Miotke-Wasilczyk (University of Gdańsk), please contact marta.miotke-wasilczyk@ug.edu.pl. The data is not publicly available.
